# Infrared Laser-Based Single Cell Permeabilization by Plasma Membrane Temperature Gradients

**DOI:** 10.3390/membranes12060574

**Published:** 2022-05-31

**Authors:** Allen L. Garner, Bogdan Neculaes, Dmitry V. Dylov

**Affiliations:** 1 School of Nuclear Engineering, Purdue University, West Lafayette, IN 47906, USA; 2 Elmore Family School of Electrical and Computer Engineering, Purdue University, West Lafayette, IN 47907, USA; 3 Department of Agricultural and Biological Engineering, Purdue University, West Lafayette, IN 47907, USA; 4 GE Research, Niskayuna, NY 12309, USA; d.dylov@skoltech.ru

**Keywords:** membrane permeabilization, microinjection, optoinjection, optoporation, temperature gradients, transfection

## Abstract

Single cell microinjection provides precise tuning of the volume and timing of delivery into the treated cells; however, it also introduces workflow complexity that requires highly skilled operators and specialized equipment. Laser-based microinjection provides an alternative method for targeting a single cell using a common laser and a workflow that may be readily standardized. This paper presents experiments using a 1550 nm, 100 fs pulse duration laser with a repetition rate of 20 ns for laser-based microinjection and calculations of the hypothesized physical mechanism responsible for the experimentally observed permeabilization. Chinese Hamster Ovarian (CHO) cells exposed to this laser underwent propidium iodide uptake, demonstrating the potential for selective cell permeabilization. The agreement between the experimental conditions and the electropermeabilization threshold based on estimated changes in the transmembrane potential induced by a laser-induced plasma membrane temperature gradient, even without accounting for enhancement due to traditional electroporation, strengthens the hypothesis of this mechanism for the experimental observations. Compared to standard 800 nm lasers, 1550 nm fs lasers may ultimately provide a lower cost microinjection method that readily interfaces with a microscope and is agnostic to operator skill, while inducing fewer deleterious effects (e.g., temperature rise, shockwaves, and cavitation bubbles).

## 1. Introduction

From the first hypotheses of medical treatments that induced genetic modification by introducing exogenous DNA, gene therapy has made enormous progress, as indicated by the first United States Food and Drug Administration (FDA) approvals for gene therapy products in 2017 [1]. At present, the most prevalent vectors are virus-based and include adenoviruses, adeno-associated viruses, and lentiviruses [2]. While promising, many challenges remain in terms of immune responses that limit in vivo administration of the vectors [1], short- and long-term safety [2,3], expense [3], and side effects [3]. This has motivated the exploration of alternatives to viral vectors for gene therapy, including electroporation and transfection with cationic polymers or lipids [3]. 

Single cell microinjection is a powerful technique used to introduce exogenous material into cells and to extract and transfer material between cells. Microinjection has been used to transfect cells, such as primary cultured human neurons and salivary gland cells. Traditional microinjection is a mechanical method that does not require the delivery of additional compounds to treated cells and largely amplifies and isolates the effects of the injected substances. Compared to other methods, microinjection provides the ability to precisely tune the volume and timing of delivery into either the cytosol or the nucleus, while requiring less of the material being delivered and exhibiting lower toxicity [4] compared to viral vectors. Microinjection can also be tuned to deliver one construct to one group of cells in a single culture dish, permitting the remaining untreated cells to serve as built-in controls under identical conditions to the treated cells. 

However, despite improvements in techniques [5], microinjection tends to be extremely labor intensive [4,6] and, therefore, expensive. This limits the ability of many research groups to employ this tool in research. Moreover, this tool has limited applicability to certain cell types (e.g., flat cells). Microinjection also requires specialized equipment and technical skills to prevent cell damage and the amount of microinjected tracer can be difficult to standardize. It is also invasive, as cell impalement can cause sudden and dramatic changes in intracellular homeostasis. Microinjection is also unsuitable for detecting rapid changes or events that require continuous application of the stimulus. 

As an example, microinjection for neurons [7] typically focuses on injecting substances that are not synthesized by cells and transfecting specific cells or cell types in a mixed cell culture. Traditional microinjection for neurons is time consuming, requires expensive equipment, induces poor survival rates due to physical damage from injection, and can have variable effectiveness depending upon neuron size and robustness [7]. One potential alternative, single cell electroporation, requires expensive equipment while also being time consuming and difficult to optimize [7]. 

To improve traditional microinjection, an alternative technique proposed to use laser beams to “perforate” cell membranes [8]. Lasers provide a physical alternative to viruses for gene therapy [9]. Researchers have performed experiments with lasers at various wavelengths, including 355 nm [10,11], 488 nm [12] or 1064 nm [13]. Most work in this field has used ≈800 nm laser wavelengths [14,15,16,17,18]. The specific mechanisms responsible for cell poration depend on the laser wavelength. Heating and thermoelastic stress are considered the dominant mechanisms at 355 nm, 488 nm, and 1064 nm, while multi-photon effects and the generation of low-density fee electron plasma are considered the dominant mechanisms at 800 nm [14]. More recent studies have moved from using CW lasers to femtosecond lasers to improve pore control and increase cell viability [14]. 

One interesting research direction proposed using a longer laser wavelength, 1554 nm [19]. In addition to providing significant practical advantages by being more compact and less expensive than 800 nm lasers, this prior work also suggested that fs lasers at 1554 nm would cause fewer deleterious effects to the transfected cells, such as “temperature rise, shock wave, and cavitation bubble generation” [19]. 

In this paper, we discuss a single cell permeabilization method using a 1550 nm fs laser (significantly less expensive than 800 nm lasers) that is easily interfaced with a microscope, user friendly, and more agnostic to (or even independent of) operator skill, while not requiring the various consumables common for conventional microinjection (e.g., special syringes, glass capillaries, and needles). This method is based on three building blocks:

1. **Visualization system.** This is an umbrella system that allows the user to (a) see the cells on a screen (such as a liquid-crystal display (LCD) monitor or a touchscreen); (b) select a cell of interest (either with the help of software or by using the touchscreen); and (c) observe the result of microinjection (e.g., morphological changes in a bright-field microscope or fluorescent nuclei of the cell imaged with an appropriate modality).
2. **Illumination/laser treatment system.** This system delivers laser energy to the cell of interest in a form that is easily absorbed by the cell (e.g., particular wavelength, beam size, and power). The laser beam creates a local temperature gradient across the cell membrane, which permeabilizes the membrane, resulting in the delivery of exogenous molecules into the cell.
3. **Registration system.** This system assures the precise placement of the laser beam spot in a known position in space. It guarantees the alignment of the visualization and illumination systems with sufficient resolution in three dimensions (X, Y, Z). Example embodiments include (separate or combined) a three-dimensional (3D) translation stage that moves the cell in all directions, various optical dispersion compensation components, fluorescent dyes, apertures, photodiodes, and cameras.

Compared to previous research [19], which strictly focused on demonstrating that a fs laser at 1554 nm could permeabilize cells to facilitate exogenous molecule delivery, the current study presents the experimental setup and challenges in performing single cell injection with IR lasers, measurements of the laser power delivered to the cells around the permeabilization threshold, and potential mechanistic pathways for cell permeabilization based on thermal gradients. In essence, we will apply the approach we previously used to calculate temperature gradients for cell permeabilization using a wide-field 1550 nm laser [20,21] to assess a single-cell, tightly focused 1550 nm laser beam. 

## 2. Materials and Methods

### 2.1. Laser Experimental Setup

**Figure 1:**
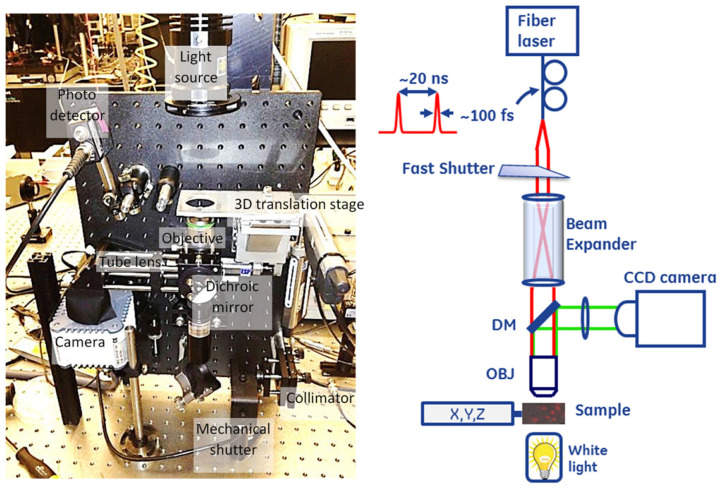
(**Left**) Photograph and (**right**) schematic of the experimental setup used for infrared (IR) single cell laser injection.

Figure 1 shows the system designed for optically driven delivery of cell material. The system was based on a conventional inverted bright-field microscope. LabVIEW (National Instruments, Austin, TX, USA) was used to integrate and control the entire setup, including visualization, registration, positioning, and turning the beam on or off. The sample was placed on a three-dimensional translation stage and illuminated with a white light source to visualize the cells. The transmitted light was collected through a microscope objective (Zeiss, White Plains, NY, USA 20×/0.8 [Inf/0.17] plan-apochromat) and reflected by the dichroic mirror (DM) before the image was projected onto the camera sensor with the tube lens (200 mm focal length). We used a Hamamatsu ORCA-ER C4742-80 camera (Bridgewater, NJ, USA) for image capture. 

The second arm of the experimental system delivered ultrafast pulse trains from a mode-locked fiber laser (Calmar, Palo Alto, CA, USA, FPL-04CFFPM, wavelength 1550 nm, pulse width 100 fs, repetition rate 50 MHz, average power > 100 mW, polarized output delivered through a Fujikura polarization-maintaining fiber) to induce cell membrane permeabilization. The laser beam was collimated with a collimator lens and expanded using a telescopic beam expander (CVI, HEBX-4.0-5X-1550, Albuquerque, NM, USA) to fill the entrance aperture of the objective lens to achieve an adequately small focus spot on the cell. We coupled the collimated laser beam into the optical path of the microscope arm through a dichroic beam splitter [Edmund Optics, Barrington, NJ, USA, part #NT69-907; reflectivity (fraction of power reflected) R > 98% at wavelength λ < 900 nm; transmissivity T > 90% at λ > 900 nm] and focused it into the field of view of the microscope through the objective lens. The objective lens used in the setup was not specifically designed for the short infrared wavelength range, causing substantial power loss on the lens interfaces while producing a reasonably tightly focused spot at the focus (1/$e^{2}$ diameter of ≈1.5 μm). A variation of this design contained a two-dimensional (2D) galvanometer scanner module inserted between the collimator and beam expander. Tilting the beam would translate into movement of the focused beam spot across the X−Y plane at the sample location. However, this necessitates a system trade-off between the magnification, which alters the laser spot size, optical power density, and ability to scan. We used an electronically controlled mechanical shutter (Thorlabs SH0.5 shutter with SC10 controller, Newton, NJ, USA), which is capable of timing the exposure above 10 ms, in the laser beam path to control the exposure dose to the laser radiation. 

For calibration purposes, the system was equipped with an InGaAs photodiode (Thorlabs-DET10D, 1200–2600 nm, 25 ns rise time, 0.8 mm^2^ area, Newton, NJ, USA) inserted into the laser beam path after the sample stage to facilitate alignment of the microscopy and laser excitation arms of the system, as described below.

### 2.2. Laser Alignment

The depth of focus of the resulting laser beam in the cell plane was estimated to be ≲10 μm for a filled objective lens. Therefore, optimizing the interaction of light with the cell membrane requires achieving this level of accuracy in focal plane positioning within the sample. The depth of focus for the Gaussian beam used here is defined as the distance around the beam waist, where the beam diverges no greater than $\sqrt{2}d_{w},$ where $d_{w}$ is the waist diameter. The system implemented in this study incorporated a method for laser alignment in both the transverse (X,Y) and axial directions.

**Figure 2:**
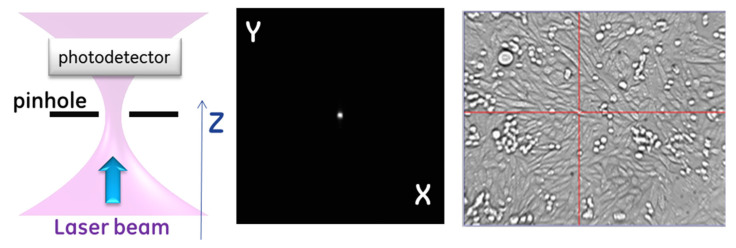
Illustration of beam alignment. (**Left**) First, the laser was aligned to pass through the 2 μm pinhole by maximizing the photocurrent on a photodetector that responds to the operating wavelength (1550 nm, InGaAs). (**Middle**) The pinhole was focused on the CCD camera and (**Right**) image as seen on the computer screen that controls the experimental setup for a test on CHO cells showing the (X,Y) position that was recorded (red cross). This method also permits optimizing Z, since high-numerical aperture (NA) objectives diverge light rapidly, and only a narrow range of Z allows passing the entire beam through the pinhole. (**Right**) Example of the user experience in selecting and permeabilizing the cell of interest by placing the red cross on the desired cell.

Figure 2 describes the system alignment. The alignment first requires registering the three-dimensional (3D) position of the laser beam waist, which we accomplished by placing a physical pinhole aperture (CVI Melles Griot, Carlsbad, CA, USA—2-micron- 04PPM001) in the field of view of the microscope near the location of the beam waist. The portion of the light transmitted through the aperture was diverted into a photodetector, which provided a measurement of the relative power passing through the aperture of the pinhole. The pinhole was placed on the 3D translation plate and moved to maximize transmission. We designed the pinhole to be close to the expected beam size to create a confocal detection condition such that the maximum signal occurred when the pinhole was in the optical conjugate point with respect to the fiber laser output aperture. For a Gaussian beam at 1550 nm with $d_{w}\approx 1.5\;\mu$m, the depth of focus was $2Z_{R}\approx 5.4$ μm, where $Z_{R}$ is the Rayleigh range, which means a ±2.7 μm axial displacement will increase the spot size by a factor $\sqrt{2}$ and the area by 2×. Matching the confocal aperture to the beam size makes the transmitted beam power through the pinhole at the edge of the Rayleigh range ≈ 73% of maximum value transmitted and ≈92% of the maximum value transmitted at $Z_{R}/2$. For the laser powers used in this experiment, changes of laser power through the pinhole on the order of a few percent are easily detected, which makes focus positioning accuracy ≲1 μm. Once the beam position was defined by the pinhole aperture, the pinhole was back-illuminated, and the microscope arm of the system was adjusted to identify the (X,Y) coordinates of the beam in the camera frame of reference and focus the image on the sample plane coincident with the focal plane of the laser. The registration of the camera focus was performed by adjusting the tube lens position with respect to the camera to achieve a sharp image of a back-illuminated pinhole. This ensured that the focusing plane of the laser beam remained fixed at the plane of the aperture (no motion of the objective lens), while the object plane of the microscope was adjusted, by small changes of magnification, to be an optical conjugate with the camera sensor plane. Thus, the object cell appearing at the (X,Y) position of the pinhole and in focus of the microscope arm was also positioned at the focal spot of the laser beam. 

There are two main reasons we could not perform beam registration by directly observing the laser beam: (1) the ultrafast laser operated in a spectral range inaccessible by silicon-based optical sensors and (2) the optical objective used for both bright-field imaging in the visible range and focusing of short-wave infrared laser light exhibited chromatic aberration across the broad spectral range. Instead, the pinhole aperture was used as a fiducial for both the laser beam (via transmitted power detection) and imaging light (via contours of the pinhole).

### 2.3. Cell Preparation

We followed the cell preparation method presented in more detail elsewhere [20]. We cultured adherent Chinese Hamster Ovarian cells [CHO, American Type Culture Collection (ATCC), Manassas, VA, USA] in F12K media supplemented with 10% FBS according to the ATCC protocol (complete media). Cells were used at an early passage, typically between passages 4 and 10. A Countess Automated Cell Counter (Invitrogen) was used for cell counts. Viability and morphology were checked 24 h after seeding, and the experiment was performed when cells were at about 30% confluence. Media was exchanged the next day using 0.5 mL F12K complete media, as described above.

### 2.4. Laser-Induced Membrane Permeabilization Assessment with Propidium Iodide

Propidium iodide (PI) stock was generated by reconstituting the powder (1 mg) in 1 mL phosphate-buffered saline (PBS) according to the manufacturer protocol (Sigma). PI was added to each experimental well of a 24-well plastic tissue culture plate at a concentration of 1 μg/mL and allowed to incubate for 5 min prior to the laser stimulation/exposure. Positive control cells were fixed and permeabilized with 4% PFA/0.1% Triton X-100 and treated with 1 μg/mL PI. Fixation and permeabilization created pores in the cell membrane that permitted the dye to enter the cells and stain them positive. Negative controls consisted of CHO cells and the PI dye incubated for the same duration without 4% PFA/0.1% Triton X-100.

We assessed PI uptake 10–15 min after laser illumination by using a Nikon Eclipse DIC phase microscope with 20 and 60 Plan Fluor objective lenses and the built-in Nikon software for image acquisition. The bright-field and cy3 images were overlaid and stitched in ImageJ software by an automated macro.

We performed a series of experiments showing cell membrane permeabilization by using average laser powers of about 15–24 mW with exposure times typically on the order of several tens of s (40 s, for instance, for the experiment reported later in the manuscript); these experiments were conducted to measure laser power when permeabilization occurred to guide the analytical model in Section 4.

## 3. Laser Permeabilization Experimental Results

**Figure 3:**
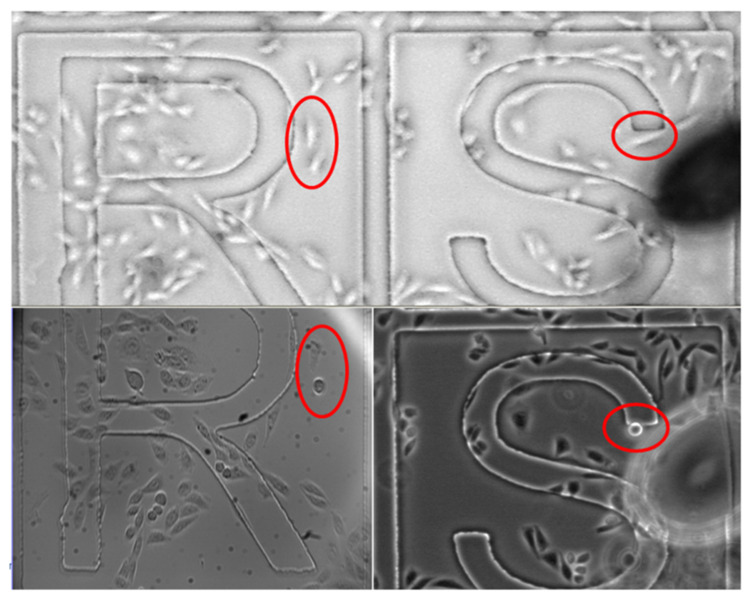
Example of CHO cells changing morphology from the expected longitudinal (circled cells in the top panels) to round shape (circled cells in the bottom panels) upon exposure to the focused laser beam (24 mW, 60 s). Each letter section above with cells has dimensions of 500 μm × 500 μm.

Before performing the permeabilization experiments, our initial tests demonstrated the ability to target cells of interest with aggressive laser exposures to later observe their morphology. Figure 3 shows a microscope image demonstrating that exposing CHO cells to an intense, focused laser beam (≈1.5 um spot) can alter them from the expected morphology/longitudinal shape to round. Figure 3 also highlights the ability to precisely target the cells of interest using this experimental setup.

**Figure 4:**
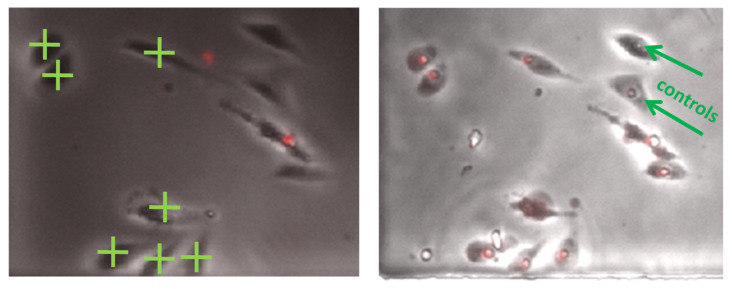
Demonstration of laser injection to 7 targeted CHO cells for 40 s of laser treatment with a peak power of 24 mW. (**Left**) Image before laser injection to the seven cells indicated by green crosses (i.e., plus signs). (**Right**) Image after laser-induced permeabilization, demonstrating propidium iodide (PI) uptake by the targeted CHO cells (denoted with the red stain) and no PI uptake by the two control cells (labeled with green arrows as controls). The physical size of these data panels is ≈275 μm × 300 μm.

We next used the laser setup to deliver PI into selected CHO cells. Figure 4 shows the delivery of PI to seven targeted CHO cells. Appendix A shows separate bright field and fluorescent photos for the results from Figure 4. Prior to laser exposure, two of the cells underwent PI uptake, suggesting that they were either already permeabilized or dead. After the laser treatment, the seven targeted cells exhibited PI uptake, while the two control cells that were outside the laser target did not. This indicates that the laser may effectively target and permeabilize specific cells without interacting with nearby neighbors. Note that while these results demonstrate our main experimental goal of effective permeabilization and PI delivery, we did not investigate viability after laser treatment; morphologically, the cells are still adherent, but they may appear impacted. Future studies can work to adjust laser parameters and treatment times to optimize delivery while minimizing adverse side effects to the treated cells.

## 4. Theoretical Interpretation of Results and Possible Mechanism for Cell Permeabilization

Previous work on IR laser single cell injection using a 1554 nm laser hypothesized the generation of free electrons or plasma formation in the lipid layer as possible mechanisms for cell permeabilization [19]. The same study further proposed that the efficiency of ionization will be much lower at 1554 nm than at 800 nm (the state of the art for most single cell laser injection work), and the resulting free electron density induced by the femtosecond laser beam at 1554 nm will be significantly reduced. The induction of membrane permeabilization with a dramatic reduction in free electron density may suggest the contribution of another mechanism for membrane permeabilization. 

Prior studies assessed the feasibility of cell membrane temperature gradients inducing or facilitating cell membrane permeabilization [21,22]. Cell membrane temperature gradients may be induced by high frequency or rapid rise-time electromagnetic radiation, most notably high-power microwaves [23,24] or fast rise-time electric pulses [21,22,25]. One experimental study demonstrated that exposing CHO cells to a 2.45 GHz microwave field induced membrane permeabilization to green fluorescent protein (GFP) with negligible temperature increase in the surrounding medium [26]; calculations of the transmembrane potential using common techniques [27,28] readily show that these fields cannot induce transmembrane potential remotely near those of conventional electroporation, which is on the order of 0.1–1 V [29], without also causing substantial bulk heating and cell death. Temperature gradients increase with faster rise and fall times and higher repetition rates [21], which would correspond to higher frequencies for alternating current (AC) fields (e.g., microwaves). We hypothesized that the cell membrane temperature gradients could cause a transmembrane voltage gradient in the polar molecules within the lipid bilayer by the thermoelectric effect that would enhance the transmembrane potential induced by the electromagnetic radiation and, consequently, membrane electropermeabilization [21]. Subsequent molecular dynamics simulations showed that adding a temperature gradient to an electric pulse could induce pore formation in a lipid bilayer that did not occur in the absence of the temperature gradient [22]. Because of the short duration of the laser pulses used in wide illumination (diameter ≈ 50 μm IR radiation), we previously assessed the feasibility of cell membrane temperature gradients as a potential mechanism for the observed cell membrane temperature gradients [30]. Despite using similar peak powers and pulse durations as standard optoinjection lasers, the power densities for the wide field illumination study were three orders of magnitude lower due to the substantially larger illumination area (≈2500 μm^2^ compared to ≈4 μm^2^ for standard optoinjection). While the prior calculations indicated that the temperature gradient induced by the wide field illumination laser was insufficient to induce electropermeabilization alone, we pointed out that the laser would at least minimally induce an increased transmembrane potential and overall sample heating that would act synergistically with the temperature gradients to enhance membrane permeabilization [30].

The present experiment uses a much narrower field of illumination (≈1.5 μm) compared to our wide field illumination study [30], decreasing the overall illumination area by a factor of ${\left(50/1.5\right)}^{2}\approx 1111$. The average power used in the present experiments (15–24 mW) is approximately 5–8× smaller than in the wide field illumination study (120 mW), making the power density here 138–222× higher than for the wide field illumination study. Thus, the average power density 〈w〉 for this experiment is between 6.6 × 10^6^ and 1.1 × 10^7^ W cm^−3^, assuming that the exposure volume is equal to the product of the illumination area and the absorption coefficient of water, which is taken to be ≈10 cm^−1^ at 1550 nm [30]. As in the prior study, the repetition rate is 50 MHz [30], making the time between individual pulses $\tau_{rep}=2\times 10^{-8}$ s. The peak energy density of a laser with a pulse duration $\tau_{p}$ is $E_{p}=\tau_{p}W_{peak}=\tau_{p}W_{avg}/\left(\tau_{p}/\tau_{rep}\right)=\tau_{rep}W_{avg},$ where $W_{peak}$ and $W_{avg}$ are the peak and average powers of the laser, respectively. This gives $0.132<E_{p}<0.22\;{J\;\mathrm{cm}}^{-3}$. Previous studies considered peak power per unit area [19], which we can obtain by $W_{peak}^{\prime }=E_{p}\tau_{p}^{-1}\delta ,$ where δ=0.1 cm is the depth of penetration from the absorption coefficient of water at this wavelength. Thus, $1.3\times 10^{11}<W_{peak}^{\prime }<2.1\times 10^{11}$ W cm^−2^ for our experiments. 

The previous theoretical assessment related the threshold for membrane potential $V_{m}$ for conventional electropermeabilization, which is typically taken as $0.1\;V≲V_{m}≲1\;V$ for a 7 nm thick cell membrane [29], to the transmembrane potential induced by a temperature gradient across the plasma membrane. Note that while many studies use 5 nm to correspond to the electrical thickness of the cell membrane [31], we choose 7 nm here to correspond to the physical dimension of the membrane [32] across which the temperature change occurs. Given the variation in the estimates of the electropermeabilization threshold and the practical differences for different cell lines [33] and waveforms [34], this difference should not introduce much additional uncertainty for an initial order of magnitude estimate. Assuming a thermoelectric conversion factor from voltage to temperature of ≈100 K/V [35] gives the corresponding range for the threshold for cell membrane temperature gradient necessary to induce electropermeabilization $\nabla T_{ep}$ as 10^9^ < $\nabla T_{ep}$ < 10^10^ K/m. 

A fixed energy density laser can induce electropermeabilization through a membrane temperature gradient by satisfying [30]

(1) $$\tau_{rep,E}<\left(E_{p}\tau_{diff}\right){\left[\rho c_{v}R\nabla T_{ep}-E_{p}{\left(\tau_{diff}/\tau_{p}\right)}^{1/2}\right]}^{-1},$$

where ρ is the mass density of the targeted material, $c_{v}$ is the specific heat of the targeted material, R is the cell radius, and the thermal diffusion time of the medium is

(2) $$\tau_{diff}\approx \frac{\rho c_{v}R^{2}}{\kappa },$$

where κ is the thermal conductivity of the cell. For a fixed average power density 〈w〉, the cell membrane temperature gradient threshold for electropermeabilization may be achieved by satisfying [30]

(3) $$\tau_{rep,\langle w\rangle }>\left(\rho c_{v}R\nabla T_{ep}-\tau_{diff}\langle w\rangle \right){\left[\langle w\rangle {\left(\tau_{diff}/\tau_{p}\right)}^{1/2}\right]}^{-1}.$$

Assuming ρ = 1000 kg/m^3^, $c_{v}=4200\;$J kg^−1^ K^−1^, R = 10 μm, and κ= 0.6W m^−1^ K^−1^ gives $\tau_{diff}\approx 7\times 10^{-4}$ s. 

**Figure 5:**
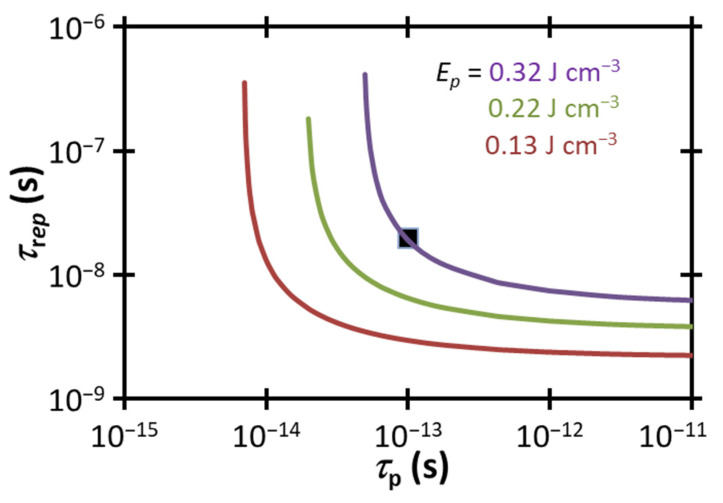
Peak energy density $E_{p}\;$to achieve $\nabla T_{ep}$, the estimated temperature gradient necessary for direct electropermeabilization, as a function of pulse duration $\tau_{p}$ and time between pulses $\tau_{rep}$. Regions below the curves, indicating a sufficiently fast repetition rate, represent $\nabla T>\nabla T_{ep}$ for the given $E_{p}$. The black square represents the experimental condition of 1550 nm, $\tau_{rep}=$ 20 ns, $t_{p}=$ 100 fs, and $0.13<E_{p}<0.22\;{J\;\mathrm{cm}}^{-3}$. Satisfying the electropermeabilization threshold for the current experimental $\tau_{p}$ and $\tau_{rep}$ would require increasing $E_{p}$ to $0.32\;{J\;\mathrm{cm}}^{-3}$.

Figure 5 compares the experimental $\tau_{rep}$ and $\tau_{p}$ to the electropermeabilization condition for a fixed $E_{p}\;$from (2) for various power densities, including the estimated experimental range of $0.13<E_{p}<0.22\;{J\;\mathrm{cm}}^{-3}$. Satisfying the temperature gradient-induced electropermeabilization threshold requires the data point to be below the line corresponding to the threshold for the given condition. The experimental combination of $\tau_{rep}$ and $\tau_{p}$ falls above the electropermeabilization condition for the potential range of $E_{p}$ in this study. One way to satisfy the electropermeabilization condition would be to increase the peak laser power $W_{peak}$ or the pulse duration $\tau_{p}$ to achieve $E_{p}\approx 0.32\;{J\;\mathrm{cm}}^{-3}$, as shown by the intersection of (2) with the data point at that value of $E_{p}$. Alternatively, we could satisfy this condition by delivering the same $E_{p}$ by adjusting $\tau_{rep}$ and $\tau_{p}.$ For the highest $E_{p}=0.22\;{J\;\mathrm{cm}}^{-3},$ we could reach the threshold by either reducing $\tau_{rep}$ to 6.53 × 10^−9^ s or decreasing $\tau_{p}$ to ≈2.9 × 10^−14^ s. Satisfying the threshold for the lowest $E_{p}=0.13\;{J\;\mathrm{cm}}^{-3}$ would require reducing $\tau_{rep}$ to ≈3 × 10^−9^ s or decreasing $\tau_{p}$ to ≈8.8 × 10^−15^ s. These calculations demonstrate the proximity of our operating conditions to this electroporation threshold and suggest the feasibility of this mechanism driving the observed permeabilization. 

**Figure 6:**
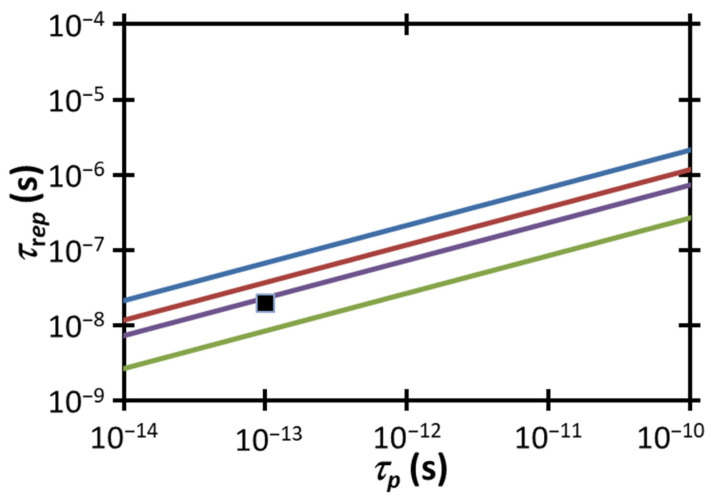
Average power density 〈w〉 to achieve $\nabla T_{ep}$, the estimated temperature gradient necessary for direct electropermeabilization, as a function of pulse duration $\tau_{p}$ and time between pulses $\tau_{rep}$. Regions above the curves, indicating a sufficiently long time between subsequent pulses, represent $\nabla T>\nabla T_{ep}$ for the given 〈w〉. The black square represents the experimental condition of 1550 nm, $\tau_{rep}=$ 20 ns, $t_{p}=$ 100 fs, and 6.6 × 10^6^ W cm^−3^ <〈w〉< 1.1 × 10^7^ W cm^−3^. Satisfying the electropermeabilization threshold for the current experimental $\tau_{p}$ and $\tau_{rep}$ would require increasing 〈w〉 to 1.6 × 10^7^ W cm^−3^.

Figure 6 compares the experimental $\tau_{rep}$ and $\tau_{p}$ to the electropermeabilization condition for a fixed 〈w〉 from (3) for various power densities, including the estimated experimental power density range of 6.6 × 10^6^ W cm^−3^ <〈w〉<1.1 × 10^7^ W cm^−3^. Unlike our wide illumination experiment, where the experimental 〈w〉 was far less than that necessary to induce electropermeabilization strictly due to a temperature gradient [31], we observe that the more focused illumination here, which dramatically increases 〈w〉, makes the selected parameters fall close to this threshold (approximately within a factor of ≈1.4–2.4×), although they are still not technically sufficient to induce electropermeabilization by the temperature gradient mechanism proposed here alone (other mechanisms, such as conventional electroporation, may contribute). Increasing 〈w〉 to 1.6 × 10^7^ W cm^−3^ by increasing the laser power or adjusting the area of the beam would achieve the thermoelectric guided electropermeabilization threshold for the experimental $\tau_{rep}$ and $\tau_{p}$. Further increasing 〈w〉 to 3 × 10^7^ W cm^−3^ provides an additional margin of safety for satisfying this condition as well as providing some flexibility in choosing a longer pulse duration to achieve the threshold. Alternatively, one could increase $\tau_{rep}$ above $3.73\times 10^{-8}$ s^−1^ (i.e., decrease the repetition rate) or decrease $\tau_{rep}$ below ≈3 × 10^−14^ s to raise the experimental data point above the limit corresponding to the experimental 〈w〉 = 1.1 × 10^7^ W cm^−3^. The lower end of the calculated range at 〈w〉 = 6.6 × 10^6^ W cm^−3^ would require increasing $\tau_{rep}$ above $6.77\times 10^{-8}$ s^−1^ or decreasing $\tau_{rep}$ below ≈1 × 10^−14^ s. As above for constant $E_{p}$, these calculations demonstrate the proximity to the electroporation threshold for plasma membrane temperature gradient-induced electropermeabilization. 

As discussed previously, this calculation only accounts for electropermeabilization induced by the transmembrane potential produced by the membrane temperature gradient by making specific assumptions about the thermoelectric effect. While the values for ∇*T* are about 1.4–2.4× lower than the threshold for temperature gradient-induced electropermeabilization $\nabla T_{ep}$, several contributory factors must also be considered. First, these calculations do not explicitly account for any laser-induced changes to the plasma membrane or any traditional electropermeabilization that may be induced by the laser’s electromagnetic field, which may cause some membrane permeabilization in an experiment even though the conditions are below the calculated threshold. Secondly, heating the cell by up to 6 K at its peak location may lower the electroporation threshold, as demonstrated by molecular dynamics simulations [36]. Initial cell permeabilization studies using a femtosecond laser at 1554 nm mention a temperature increase of approximately 7 K [19]. Therefore, one would expect a lower electroporation threshold in our experiments compared to the initial calculations included in this section. Additionally, variations in cell membrane thickness, density, and thermal conductivity may introduce additional uncertainty regarding the thermal gradient threshold for electroporation. These uncertainties coupled with the reduced electroporation threshold due to modest heating of the extracellular fluid based on our laser parameters suggest that plasma membrane thermal gradient-induced electropermeabilization may be a key mechanism for the permeabilization observed here and in previous work [19]. 

## 5. Conclusions

We have shown that the selective laser-based delivery of PI to individual CHO cells using a 1550 nm, 100 fs duration laser with average powers of approximately 15–24 mW can be explained by comparing to a simple theory relating the induced plasma membrane temperature gradient to the electropermeabilization threshold based on the thermoelectric effect. The experiments here were designed to demonstrate a system embodiment and measure the laser power necessary to permeabilization the cells. Although room remains for parameter optimization, the ability to selectively target individual cells provides exceptional utility for research studies where it is paramount to maintain identical conditions between exposed and control cells and for therapies requiring delivery specifically to individual cells or cell types. From a practical perspective, the 1550 nm laser used here is more compact and less expensive than the 800 nm lasers commonly used for optoinjection, which facilitates the introduction of this approach into clinical workflows. While another study examined 1554 nm lasers for optoinjection [19], it did not specifically detail the experimental setup and its challenges (illumination/laser treatment, visualization, registration), how the laser power was delivered to the cells (presumably directly at individuals cells given the reported 2 μm diameter of the laser focus), how much laser power was delivered to the cells, or the proposed mechanism aside from comparing the power density to that commonly reported in the optoporation literature [37]. The experiments reported here demonstrate the system alignment, automation, and workflow of a system that may be optimized to selectively permeabilize individual cells. Future studies may be performed for cell lines of interest to optimize performance by adjusting various experimental parameters, such as vertical alignment (cf., Figure 2), laser power, exposure time, and the concentration of the molecule delivered (e.g., He et al. used higher concentrations of PI [19] than we do in this study, which may facilitate significantly higher delivery efficiency at higher material cost). 

We point out that while we have not explicitly studied viability here, an eventual future instrument based on the approach described here would require tuning device parameters to optimize delivery efficiency while maintaining cell viability. The peak power density (per unit area) for our laser ranged from $1.3\times 10^{11}<W_{peak}^{\prime }<2.1\times 10^{11}$ W cm^−2^, which is within an order of magnitude of the peak power density (10^12^ W cm^−2^) reported in a prior study that observed no loss of short-term viability or apoptosis after fs laser-induced transfection [19] with similar wavelength. In this prior experiment [19], only some fraction of this total power will reach the cells, making the peak power density at the cell level < 10^12^ W cm^−2^. Since our treatment times are similar to theirs (up to 20 s for theirs [19], 40 s for ours) and we reasonably assume they succeeded in delivering $10^{11}<W_{peak}^{\prime }<10^{12}$ W cm^−2^ to the cells, we anticipate similar behavior concerning viability (loss of viability may be expected with peak power densities that exceed that reported in [19]). Thus, our experimental observations and this direct comparison may suggest minimal loss of viability, although a future parametric study is warranted to assess this across a wider range of laser exposure parameters, treatment times, beam vertical alignment, molecules for delivery, and cell lines. 

System development would be aided by a more detailed exploration of theoretical mechanisms. Theoretical calculations indicate that electropermeabilization induced by plasma membrane thermal gradients may be a significant mechanism for this laser-induced microinjection. Current studies are linking electroporation models [38] to temperature gradient calculations for electric pulse conditions, which may be relevant here for incorporating time-dependent phenomena. Future studies may also examine the transmembrane potentials induced by the applied lasers and attempt to apply these approaches to standard electroporation models [38] or to molecular dynamics simulations [22] to elucidate the mechanisms involved. Furthermore, the theory developed here is critical for understanding the interaction between lasers with these wavelengths and biological samples for various biomedical and life science applications, such as exogenous molecule delivery at 1554 nm [19], corneal surgery at 1650 nm [39], cell fusion at 1550 nm [40], and laser manipulation of human eggs and embryos from 1450 to 1480 nm [41].

In summary, laser-based microinjection using 1550 nm lasers is a potentially inexpensive tool for targeted molecular delivery in cell-based therapies that would also be relatively agnostic to operator skill, while expected to induce fewer deleterious effects, such as temperature rise, shockwaves, and cavitation bubbles, than the 800 nm lasers in use today [19]. Future studies optimizing system parameters and design for different cell lines may provide further insight into the potential long-term applications of this technology. 

## Figures and Tables

**Figure A1:**
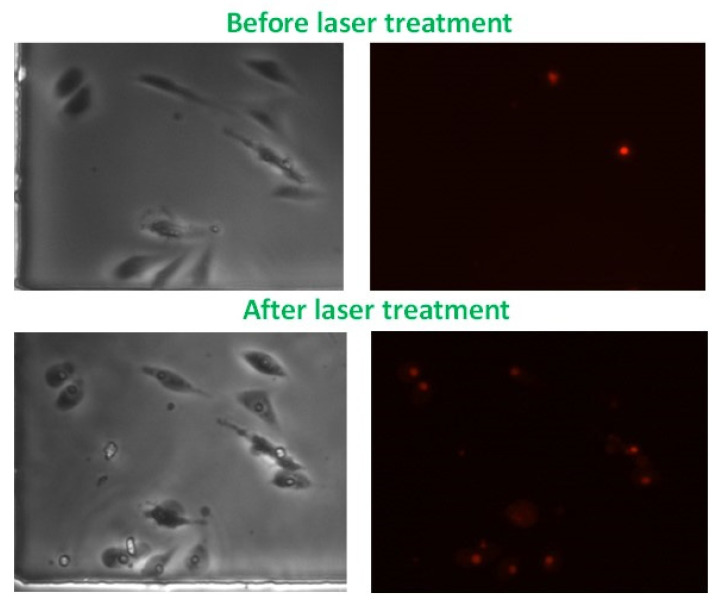
Images from Figure 4 in separate bright field and fluorescent photos.

## Data Availability

The data presented in this study are available in the article.

## Appendix A

Bright field and fluorescent images of the experimental results from Figure 4 before and after laser exposure are shown in Figure A1.
